# Longitudinal Data to Enhance Dynamic Stroke Risk Prediction

**DOI:** 10.3390/healthcare10112134

**Published:** 2022-10-27

**Authors:** Wenyao Zheng, Yun-Hsuan Chen, Mohamad Sawan

**Affiliations:** 1 CenBRAIN Neurotech Center of Excellence, School of Engineering, Westlake University, Hangzhou 310024, China; 2 Institute of Advanced Study, Westlake Institute for Advanced Study, Hangzhou 310024, China

**Keywords:** stroke, longitudinal data, backward joint model, prediction, prevention

## Abstract

Stroke risk prediction based on electronic health records is currently an important research topic. Previous research activities have generally used single-time physiological data to build static models and have focused on algorithms to improve prediction accuracy. Few studies have considered historical measurements from a data perspective to construct dynamic models. Since it is a chronic disease, the risk of having a stroke increases and the corresponding risk factors become abnormal when healthy people are diagnosed with a stroke. Therefore, in this paper, we applied longitudinal data, with the backward joint model, to the Chinese Longitudinal Healthy Longevity and Happy Family Study’s dataset to monitor changes in individuals’ health status precisely on time and to increase the prediction accuracy of the model. The three-year prediction accuracy of our model, considering three measurements of longitudinal parameters, is 0.926. This is higher than the traditional Cox proportional hazard model, which has a 0.833 prediction accuracy. The results obtained in this study verified that longitudinal data improves stroke risk prediction accuracy and is promising for dynamic stroke risk prediction and prevention. Our model also verified that the frequency of fruit consumption, erythrocyte hematocrit, and glucose are potential stroke-related factors.

## 1. Introduction

Strokes are the leading cause of death in China, leading to an expensive economic burden of over RMB 40 billion per year [1]. According to the “*Brief report on stroke prevention and treatment in China, 2020*”, there are approximately 2.8 million new stroke patients in China every year [2]. Fortunately, over 75% of strokes are preventable by controlling metabolic and behavior risk factors [3]. Among these risk factors, high blood pressure, cigarette smoking, and cholesterol imbalance, etc., are avoidable [4]. Various questionnaires and tools are proposed to predict the risk resulting from the combination of these reported risk factors. For example, the latest QRISK3 risk prediction algorithm was developed to estimate the 10-year cardiovascular disease risk in the United Kingdom [5]. However, these assessments only include present physiological parameters, attaining a static model that can only represent health status at a given time point. Even when considering the historical data in the prediction models, only the diagnosis of common diseases, e.g., diabetes and atrial fibrillation, or medication history, is considered. However, stroke is a chronic disease, so physiological information changes, and these variations can result in different stroke risk stratification [6]. Hence, we propose using longitudinal dynamic risk factors.

The concept of longitudinal data is continuously duplicated measurements on the same feature during a specific period, which reflect the variation in the feature [7]. In previous studies, longitudinal data have been used primarily to monitor the lesion progression of cognitive problems and neurological diseases, rather than prevention. Moreover, the prediction features were mainly images or disease-related biomarkers that required extra examination, rather than physical routine examination data or electronic health records (EHRs). For example, in the study on functional changes during working memory in Huntington’s disease, Poudel et al. (2015) compared fMRI activity images over 30 months [8]. In another study to predict the final outcome of primary biliary cholangitis, serum bilirubin, albumin, and prothrombin were used as longitudinal data [9].

We found that the application of longitudinal data to predict cardiovascular diseases is limited. Zhao et al. (2019) considered seven years of observations of cholesterol, body mass index, blood pressure, smoking status, and other risk factors for cardiovascular disease prediction. They exploited the temporal information by dividing the entire observation into one-year slice windows and calculated the median, maximum, minimum, and standard error for each feature, within each slice window. However, for the same features in different slice windows, the authors treated them individually, regardless of changing trends over the years [10]. This means their methods cannot reflect the impact of feature dynamic changes on risk. Therefore, in this paper, we predicted the risk of having a stroke using risk factors’ variation trends. In addition, the current situation regarding strokes in China is serious, but fewer models are based on the Chinese elderly. According to this, the Chinese Longitudinal Healthy Longevity and Happy Family Study’s (CLHLS-HF) dataset [11,12], which contains abundant physiological and life habit information, was selected in this research to construct an efficient prediction model that focused on this group of people.

From a model perspective, the previous stroke prediction models can be divided into two categories. One is based on statistical models that use mathematical equations to present stylized expressions of the relationship between the factors and the prediction results. The shortcoming of these models is the relatively low prediction accuracy [13]. One recent example is the SCORE2-Older Persons algorithm, which estimates the 5- and 10-year risk of cardiovascular disease in people over 70 years of age, yielding c-indices from 0.63 to 0.67 [14]. Another popular prediction model is applying machine-learning algorithms that can handle high-dimensional features to attain remarkable prediction accuracy. For instance, Singh and Choudhary (2017) combined a decision tree, a principal component analysis, and a back-propagation neural network algorithm to predict the results, with 97.7% accuracy [15]. However, the disadvantage here is that these algorithms do not depend on the rule designed, so the relationships between factors and outcomes are indistinct and difficult to explain. Moreover, overfitting is a frequent drawback of these machine-learning models.

Apart from commonly used stroke risk prediction scales, the purpose of most recent publications regarding stroke risk prediction has been algorithm validation. Mostly, only the overall accuracy, sensitivity, and specificity of the models are reported [16,17]. Detailed examples are rarely given to illustrate their practical applications and the feedback a patient can receive. Therefore, we propose a solution to present the dynamic results, providing information in the form of an individual dynamic prediction plot and the corresponding risk level.

In this paper, we have presented a dynamic stroke risk model using longitudinal data. Compared to prior research activities, which are considered single measurements, this is a new direction for improving prediction accuracy by monitoring historical health records. From the model’s perspective, a new algorithm, named the backward joint model, and some attempts to fit the dataset, including another variance estimation method and validation, were implemented. To the best of our knowledge, this is also the first time that this model has been applied to a complex dataset with preprocessing work. To construct a model specifically for the Chinese elderly population, the CLHLS–HF dataset was applied in this research. Moreover, our model also used Random Forest–Recursive Feature Elimination (RF-RFE), a machine-learning algorithm used to determine infrequently used factors. The individual dynamic prediction plot indicates the variation in the risk of having a stroke over the period of longitudinal data collection. This information can be further implemented in the user interface, e.g., mobile applications, to provide the latest feedback on the risk of having a stroke as well as its changes over time. Moreover, the resulting risk-of-stroke values alert people to act according to the risk value and category they are assigned. In the future, the model can be applied to multimodal data and wearable devices for real-time monitoring and can be promoted for more users and scenarios.

The remainder of this paper includes Section 2 and Section 3, in which we describe the methods and results of the data preprocessing, model construction, and prediction performance. In Section 4, we discuss the results based on the dataset and the model and give some comparisons with other related studies. Conclusions are given in Section 5.

## 2. Materials and Methods

The dataset and prediction model applied in our study are introduced in Section 2.1 and Section 2.2, respectively.

### 2.1. Dataset

**Figure 1:**
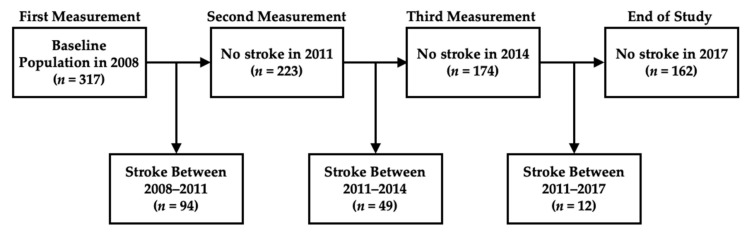
Flowchart of the observation timeline, where *n* indicates the number of individuals.

The dataset was obtained from the Chinese Longitudinal Healthy Longevity and Happy Family Study (CLHLS-HF), collected by the Centre for Healthy Aging and Development Studies of the National School of Development, at Peking University [11,12]. This open dataset is available for personal academic and policy research activities. The dataset is composed of two parts. The first part is a questionnaire containing sections of basic information, as follows: personality, mini-mental state examination (MMSE), lifestyle, activities of daily living and instrumental activities of daily living, personal background, objective examination, and illness diagnosis. The second part consists of biomarkers, with blood and urine routine test information, e.g., blood cell count and urine microalbumin. Each question and the recorded data on each physiological parameter, are considered as one factor. Overall, there are over 300 parameters in this dataset. We used the latest available dataset, including the investigations between 2008 and 2017, as shown in Figure 1.

Specifically, first, only individuals without a stroke, identified before 2008, were included in our study. Next, for everyone, the same factors were measured three times: in 2008, 2011, and 2014, with three-year gaps between each measurement. Since the data were collected by the social science department, with wide coverage and a long time interval between each measurement, we could only use these three longitudinal data points to construct the prediction model. The end time point of the study was 2017, at which point the final stroke status was collected, therefore, defined the limit of the prediction results.

The data cleaning started with the adaptation of the dataset to the model-required format, then family and post-stroke information were excluded, as this information is not relevant to an individual’s first stroke prediction. Referring to an earlier study, personality and mini-mental state examination (MMSE) [18] were located according to the score and four corresponding levels: normal, mild disabilities, moderate disabilities, and severe disabilities.

Longitudinal data show distinct superiority over the traditional single measurement in imputing missing data since they contain personal history. Hence, more information can be consulted to accurately estimate the missing value. The imputation methods can be generalized into two types, according to different data categories. One type is categorical data—a collection of information that is divided into groups, such as disease history and living area. The imputations were achieved by referring to the historical data of the individual subject. For those that cannot be imputed by the existing data, the corresponding proportion for each level was used, e.g., if one feature had two categories with 80% and 20%, then these two categories became 4:1, randomly imputed to missing individuals [19]. The proportion of the missing data imputed using this approach was up to 5%. Another type is numerical data, which is in the form of numbers, such as the value of blood biomarkers. Because of the complexity of this type, machine-learning methods are preferred for missing data imputation. Personal history and others with the same gender and age were considered to contribute to the imputation. This was achieved in our study by the multiple imputation (MI) algorithm, with a small mean square error and effective imputation efficiency [20]. For this type of data, the missing data proportion of each factor is approximately 5–18%. It has been proven that MI can still produce reasonable imputation results in this case [21].

Concerning feature selection, in prior publications, predictors were commonly restricted to preselected, established risk factors. Since the backward joint model was proposed to accommodate multiple longitudinal data, a factor increment was considered by machine-learning algorithms. Random Forest–Recursive Feature Elimination (RF-RFE) was the preferred technique for examining all potential feature subgroups and finding the best subgroup, with fewer features, to achieve the highest classification accuracy [22]. Feature importance was also calculated for confirmation.

### 2.2. Model Implementation

The proposed model for our dataset was adapted from the backward joint model (BJM) [9], with the advantage of resolving the issue that ordinary joint models must contain time-to-event data, and of supporting the consideration of censored data for model construction. Furthermore, it is computationally simpler, as it always has a one-dimensional integral in the time domain.

Here are some notations for the model. The individual is indexed by *i*, the measurement is indexed by *j*, and the main numerical factor is indexed by *g*. $Y_{ig}$ denotes the value of main numerical factors, and $Z_{ig}$ denotes the value of remaining factors. The measurement time point is denoted by $t_{ij}$, which, in our study were 0, 3, and 6. ${\tilde{T}}_{i}$ is the time-to-event data, which, in our study was the time point identified as having had a stroke and may have a value of 3, 6, or 9. $C_{i}$ is the censored time, equaling the time point at which the final status of being a no-stroke patient was recorded at 9 in our study. $T_{i}$ = min (${\tilde{T}}_{i}$, $C_{i}$), represents the time point when it comes first, and $\delta_{i}=1\left\{{\tilde{T}}_{i}\leq C_{i}\right\}$ is the event indicator, indicating whether a stroke was identified inside the follow-up time. This model assumes that $C_{i}$ is independent of ${\tilde{T}}_{i}$, $Y_{i}$ is conditional on $Z_{i}$, $t_{ij}$ is conditionally independent of $Y_{i}$ and conditional on ${\tilde{T}}_{i}$, $C_{i}$, and $Z_{i}$. The model can be summarized as follows: 

The aim of this model is to predict the risk of stroke for a new subject, *o*, within a pre-defined prediction horizon, as shown in the following equation:

(1) $$P\left(s<{\tilde{T}}_{o}\leq s+△|\bar{Y_{o}\left(s\right)},\;T_{o}>s,\;Z_{o}\right)=\frac{P\left(\;\bar{Y_{o}\left(s\right)},\;s<{\tilde{T}}_{o}\leq s+△,T_{o}>s|\;Z_{o}\right)}{P\left(\bar{Y_{o}\left(s\right)},T_{o}>s|\;Z_{o}\right)}\;=\frac{P\left(\;\bar{Y_{o}\left(s\right)},\;s<{\tilde{T}}_{o}\leq s+△|\;Z_{o}\right)}{P\left(\;\bar{Y_{o}\left(s\right)},{\tilde{T}}_{o}>s|\;Z_{o}\right)}$$

where *s* is the time point at which the prediction is made, and △ is the pre-defined prediction horizon—three-year in our study. $\bar{Y_{o}\left(s\right)}$ and $Z_{o}$ are the longitudinal history information of main numerical factors and remaining factors until *s*. The second equality is due to the assumption that $C_{o}$ is independent. For both the denominator and numerator of Equation (1), their probabilities can be easily calculated with conditional joint distribution $f(Y,\tilde{T}|Z)$, which can be decomposed into two conditions: $f(Y|\tilde{T},Z)$ and $f(\tilde{T}|Z)$. For survival sub-model $f(\tilde{T}|Z)$, we used a Cox proportional hazard model, with piecewise constant baseline hazard function. For $f\left(Y|\tilde{T},Z\right)$, we used multivariate linear mixed models, such as the following:

(2) $$y_{ig}\left(t_{ij}\right)=\beta_{0g}+Z_{i}^{T}\beta_{1g}+{\tilde{T}}_{i}\beta_{2g}+t_{ij}\beta_{3g}+{\tilde{T}}_{i}t_{ij}\beta_{4g}+\gamma_{i0g}+t_{ij}\gamma_{i1g}+\epsilon_{ijg}\;=X_{ig}^{T}\left({\tilde{T}}_{i},Z_{i},t_{ij}\right)\beta_{g}+A_{ig}^{T}\gamma_{ig}+\epsilon_{ig}$$

This equation was specified for each main numerical factor, at each measured time point, with its own parameters. It was intended to introduce the association between main numerical factors and remaining factors and time. The interaction in fixed effects was considered only between ${\tilde{T}}_{i}$ and $t_{ij}$, and $t_{ij}$ was also treated as the random effect. Equation (2) indicates that given remaining factors’ covariates, subjects with different survival times will have different main numerical factor trajectories. It can be further generalized to the second equality. After these, the multivariate linear model for all the longitudinal biomarkers was as follows:

(3) $$Y_{i}|{\tilde{T}}_{i},\;Z_{i},\gamma_{i}=X_{i}\left({\tilde{T}}_{i},\;Z_{i},t_{i}\right)\beta +A_{i}\gamma_{i}+\epsilon_{i}$$

where $X_{i}\left({\tilde{T}}_{i},\;Z_{i},t_{i}\right)$ and $A_{i}$ denote block diagonal matrices of fixed and random effects for main numerical factors, respectively. $t_{i},\;\beta$, $\gamma_{i}$ are the concatenated vectors of feature measurement time points, and fixed and random coefficients. $\epsilon_{i}$ is the specific measurement error. $\gamma_{ig}={\left(\gamma_{iog},\;\gamma_{i1g}\right)}^{T}$ is assumed to have a multivariate normal distribution, as follows:

(4) $$\mathrm{MVN}\left(0,\;\;\Omega_{gg}=\left(\begin{array}{cc} \sigma_{\gamma_{0g}}^{2}\left({\tilde{T}}_{i}\right) & \sigma_{\gamma_{0g}}\sigma_{\gamma_{1g}}\left({\tilde{T}}_{i}\right)\\ \sigma_{\gamma_{0g}}\sigma_{\gamma_{1g}}\left({\tilde{T}}_{i}\right) & \sigma_{\gamma_{1g}}^{2}\left({\tilde{T}}_{i}\right) \end{array}\right)\right)$$

and $\epsilon_{i}$ is assumed to have normal distribution: $N\left(0,\;\sigma_{\epsilon_{g}}^{2}\left({\tilde{T}}_{i}\right)\right)$, with both variance and covariance parameters dependent on ${\tilde{T}}_{i}$. The possible relationships between the different main numerical factors are presented by the correlation between their random effects, i.e., the covariance between $\gamma_{ig_{1}}$ and $\gamma_{ig_{2}}$ is denoted by $\Omega_{g_{1}g_{2}}\left({\tilde{T}}_{i}\right)\left(g_{1}\neq g_{2}\right)$.

The denominator and numerator of Equation (1) were calculated similarly, the only difference being that $s<{\tilde{T}}_{o}\leq s+△$, or ${\tilde{T}}_{o}>s$. For example, the denominator can be decomposed as follows:

(5) $$P\left(\;\bar{Y_{o}\left(s\right)},{\tilde{T}}_{o}>s|\;Z_{o}\right)=\overset{\infty }{\underset{s}{\int }}f(\bar{Y_{o}\left(s\right)}\;|{\tilde{T}}_{o}=u,\;Z_{o})f\left({\tilde{T}}_{o}=u,|Z_{o}\right)du$$

To properly integrate the entire support of ${\tilde{T}}_{o}$, we assumed that ${\tilde{T}}_{o}$ was within $C_{o}$, given $Z_{o}$. However, approximately half of the individuals in our study were categorized as no-stroke patients during this period, especially for future application to new arrivals, those without ${\tilde{T}}_{o}$. Therefore, the present algorithm used a two-part model, involving verifiable assumptions for this group of subjects, with $\tilde{T}$ larger than a pre-specified maximum follow-up time, τ, which, in our study, was 9 years. This group of patients with $\tilde{T}>\tau$ were referred to as long-term survivors (LTS), and Equation (3) was modified to the following:

(6) $$Y_{i}|Z_{i},{\tilde{T}}_{i}>\tau ,\;\gamma_{i}=X_{i}{}^{e}\left(Z_{i},t_{i}\right)\beta^{e}+A_{i}{}^{e}\gamma_{i}{}^{e}+\epsilon_{i}{}^{e}$$

The superscript *e* was used to distinguish the LTS group, which differs from Equation (3) in that $T_{i}$ was not added as an effect to Equation (6). This equation characterizes the longitudinal trajectory of a heterogeneous group of subjects, with different survival times, $\tilde{T}$ > τ. Therefore, the two-part model for $f\left(Y|\tilde{T},Z\right)$ can be summarized as follows:

(7) $$Y_{i}|{\tilde{T}}_{i},\;Z_{i}\sim \mathrm{MVN}\left(X_{i}\left({\tilde{T}}_{i},\;Z_{i},t_{i}\right)\beta ,\;V_{i}=A_{i}\Omega A_{i}{}^{T}+\Sigma_{i}\right)\;\;\;\;\;\;\;\;\;\;\;\left({\tilde{T}}_{i}\;\leq \;\tau \right)$$

(8) $$Y_{i}|{\tilde{T}}_{i},\;Z_{i}\sim \mathrm{MVN}\left(X_{i}{}^{e}\left(Z_{i},t_{i}\right)\beta^{e},\;V_{i}{}^{e}=A_{i}{}^{e}\Omega^{e}A_{i^{e}}{}^{T}+\Sigma_{i}{}^{e}\right)\;\;\;\;({\tilde{T}}_{i}>\tau )$$

where *MVN* is multivariate normal distribution, Ω and $\Omega^{e}$ denote the covariance structure, combining $\Omega_{gg}$ and $\Omega_{g_{1}g_{2}}\left(g_{1}\neq g_{2}\right)$. $\Sigma_{i}$ and $\Sigma_{i}{}^{e}$ denote the measurement error variance.

In the estimation procedure, a two-stage pseudo-maximum likelihood estimation procedure was proposed to fit BJM. The first stage was to fit the survival model of $f(\tilde{T}|Z)$ and obtain maximum likelihood estimators of its parameters. Next, the second stage was to fit longitudinal models of $f\left(Y|\tilde{T},Z\right)$ by expectation–maximization (EM) algorithm, which is divided into E-step and M-step. During the EM iterations, the survival model parameters were fixed to their first-stage estimators. Both the random effects, $\gamma_{i}$, and the unobserved, ${\tilde{T}}_{i}$, were dealt with in this process. The coarsening approximation [23] was used here to represent the residual lifetime distribution of $T_{i}$ after the censoring time. It replaced the continuous distribution of $\tilde{T}$ with a discrete mixture distribution, facilitating the calculation of the EM algorithm. 

In the E-step after *m*th iteration, we calculated the conditional expectation for $\gamma_{i}$ and $\gamma_{i}\gamma_{i}{}^{T}$, with known $Y_{i},\;\tilde{T_{i}},\delta_{i}$ and ${\hat{\Theta }}^{\left(m\right)}$, based on the conjugate prior in Bayes’ rule. Similar process was also performed for the LTS model. Both $f(\tilde{T}|Z)$ and $f\left(Y|\tilde{T},Z\right)$ were required here to calculate the conditional expectation of $\left\{{\tilde{T}}_{i}=l_{ik}\right\}$ and $1\{{\tilde{T}}_{i}>\tau \}$.

In the M-step, we computed the maximum expectation of complete data log-likelihood, which was also the final derivation of Equation (5), as follows:

(9) $$\overset{n}{\underset{i=1}{\sum }}(\delta_{i}\log(f(Y_{i}|{\tilde{T}}_{i}=T_{i},\;Z_{i},\gamma_{i};\Theta )P\left(\gamma_{i};\Theta \right))\;+\left(1-\delta_{i}\right)\overset{K}{\underset{k=1}{\sum }}(1\left\{{\tilde{T}}_{i}=l_{ik}\right\}\log(f(Y_{i}|{\tilde{T}}_{i}=l_{ik},\;Z_{i},\gamma_{i};\Theta )P\left(\gamma_{i};\Theta \right))\;+\left(1-\delta_{i}\right)1\{{\tilde{T}}_{i}>\tau \}\log(f(Y_{i}|{\tilde{T}}_{i}>\tau ,Z_{i},\gamma_{i}{}^{e};\Theta^{e})P\left(\gamma_{i}{}^{e};\Theta^{e}\right)))$$

where Θ={β, Ω, σ} and $\Theta^{e}=\left\{\beta^{e},\Omega^{e},\sigma^{e}\right\}$, $l_{ik}$ is the stroke time point calculated by coarsening approximation. K was assigned a value of 10 here, as it has been proved to produce sufficient approximation and the result was not sensitive to further increase in K [23]. $P\left(\gamma_{i};\Theta \right)$ and $P\left(\gamma_{i}{}^{e};\Theta^{e}\right)$ are the density function for individual random effects. $f(Y_{i}|{\tilde{T}}_{i}=s,\;Z_{i},\gamma_{i};\Theta )$ and $f\left(Y_{i}|{\tilde{T}}_{i}>\tau ,Z_{i},\gamma_{i}{}^{e};\Theta^{e}\right)$ are from Equations (3) and (6). To maximize the expectation, after *m*th iteration, the parameters in Equation (9) were replaced by {${\hat{\Theta }}^{\left(m\right)}$, ${{\hat{\Theta }}^{e}}^{\left(m\right)}$}. After the EM algorithm converged at the end of the iterations, we obtained the denominator result of Equation (1). The numerator of Equation (1) was calculated by the same procedure. Finally, we obtained the result of Equation (1), i.e., the expected stroke risk prediction result.

Since this paper focused, not on algorithm construction, but on the application of this novel model to stroke prediction, we only summarized the broad construction steps here. Detailed equation derivations can be found in BJM proposed paper [9].

The original model, proposed by Shen and Li, used 300 bootstrap repetitions for variance estimation, resulting in a large confidence interval [9]. Therefore, another commonly used method, repeated 10-fold cross-validation [24], was also attempted for bias and variance comparison in our work. Data preprocessing, analysis, model construction, and validation were all performed using R on RStudio (version 4.1.1 for Windows 10, RStudio, PBC., Boston, MA, USA).

## 3. Results

### 3.1. Baseline Characteristics

The outcomes of stroke status in our prediction model were “no stroke” and “identified as having had a stroke”, which were defined by either being diagnosed by medical doctors in the hospital or self-reporting (a “yes” or “no” status). These two questions regarding stroke identification were included in the questionnaire. After excluding individuals lost in the study and those with an unclear stroke status, 317 participants were included in 2008, aged between 62 and 105 years. In all, 94 were identified as having had a stroke between 2008 and 2011, 49 were identified as having had a stroke between 2011 and 2014, and 12 were identified as having had a stroke between 2014 and 2017. The remaining 162 did not suffer a stroke before the end of the observation period (Figure 1). In summary, 714 measurement records were used to construct the prediction model. 

With the completion of the preprocessing, the total number of predictors was reduced from over 300 to 90 after we excluded their relatives and post-stroke information. In our prediction model, the 20 most stroke-related factors were considered. Ten were established factors, and ten others were determined using RF-RFE, as introduced in Section 2.1. The established factors used in this paper were defined by a guideline named “*American College of Cardiology/American Heart Association (ACC/AHA) guideline on assessment of cardiovascular risk*” [25] and have also been widely used by other stroke risk calculators [5,26]. These features include systolic blood pressure, diastolic blood pressure, total cholesterol, high-density lipoprotein cholesterol, smoking, sex (male/female), age, province (south/north), geographic location (rural/urban/town), and diabetes history (yes/no). The RF-RFE features of choice are red cell count, platelet count, erythrocyte hematocrit, blood urea nitrogen, hemoglobin, glucose, frequency of doing housework (every day/at least once a week/at least once a month/sometimes/never), frequency of fruit consumption (almost every day/quite often/occasionally/rarely/never), mini-mental state examination (MMSE) (normal/mild/medium/severe), and hypertension history (yes/no). These 20 factors can also be divided into main numerical factors and remaining factors. 

First, the main factor must be numerical data, as categorical data do not have continuous numerical meanings. Next, for these 12 numerical factors, the main numerical factors must fulfill at least one condition for its value or variation trend to distinguish the stroke group from the no-stroke group clearly. The Welch t-test was used to compare the differences between these two groups, and, finally, the *p*-value < 0.05 defined the statistically significant difference [27]. In the numerical value comparison, we compared the value of the initial features in 2008 for patients who were identified as having had a stroke before 2011, and for those who did not have a stroke until 2011. The same was then undertaken for the following years so that three *p*-values could be obtained for each feature. The grouping here was based on the differences in single measurement. Systolic blood pressure, total cholesterol, high-density lipoprotein cholesterol, platelet count, and age showed significance at least once, while the remaining features were insignificant in all three *p*-values. In comparing the variation trends, we calculated two variation values between 2008 and 2011, and between 2011 and 2014 for the same feature, for everyone, so that two *p*-values could be obtained for each feature. The grouping here was based on the final status in 2017 because we believed that the stroke and the no-stroke patients would have different longitudinal trajectories of risk factors. Therefore, these comparisons focused more on the differences in dynamic changes.

Systolic blood pressure, diastolic blood pressure, and red blood cell count showed significant differences between the stroke and no-stroke groups. In contrast, other factors still did not show any significant difference. The full *p*-value table can be found in the Supplementary Materials (Table S1). Based on the two comparisons above, seven factors appeared to be significantly different between the two groups. Although age showed one significant difference in the first measurement (2008), the individual variation slope was the same, and it was also the same as the variation in measurement time, so we did not consider it as a main numerical factor. After this consideration, the rest of the factors—systolic blood pressure, diastolic blood pressure, total cholesterol, high-density lipoprotein cholesterol, red blood cell count, and platelet count—were defined as the main numerical factors. The remaining were treated as remaining factors with categorical factors.

The average and standard deviation of the six main predictors at each measurement are summarized in Table 1. Table 2 summarizes the characteristics of the remaining 14 predictors. All the patients were divided into a stroke group or a no-stroke group. The number of no-stroke patients (not identified as having had a stroke before the end of the study) was 162, and we used Equation (2). The other group comprised stroke patients and the total number changed because patients’ longitudinal data collection stopped after the stroke onset. More specifically, 2008 saw 155 patients, including all those who were identified as having had a stroke during the study. In 2011, there were 61 patients, as 94 patients were identified as having had a stroke in 2008–2011, so their measurements were stopped. In 2014, there were only 12 stroke patients, as 49 patients were identified as having had a stroke between 2011 and 2014 and their measurements were ended.

### 3.2. Longitudinal Biomarker Equations and Relationships with Other Risk Factors 

Based on Equations (3) and (6), Equations (10)–(21) were constructed for each of the main numerical factors. The initial model contained all the remaining factors. Next, we used the analysis of variance to calculate the F-values and *p*-values to determine the significance of the remaining factors. Considering that the significance of the categorical factors can vary in different categories, we also referred to the t-values and their corresponding *p*-values, which were obtained by the R function, “lme”. If one category of a categorical factor had a significant effect, we also considered this factor in the final model. The R function, “regsubsets”, considered multiple model selection criteria together and was also used to find all the best possible models. Finally, we decided on the final model by referring to the coefficients, standard errors, and *p*-values. The *p*-values for the last selected features were all less than 0.1, and the final model included as many factors as possible, as we were also interested in the relationship between the main numerical factors and the remaining factors. All features were expressed as abbreviations, and the different numbers indicate the levels of categorical data. The corresponding details can be found in Table 2. 

The difference between the stroke and LTS groups was that LTS group individuals did not have a stroke-identified time point (${\tilde{T}}_{i}$). The following are equations based on the stroke individuals’ longitudinal data, where *i* denotes the individual and *j* denotes the measurement time point:

(10) $$\begin{array}{ll} \mathrm{Systolic Blood Pressure} & =\beta_{01}+{\tilde{T}}_{i}\beta_{11}+{\mathrm{hct}}_{i}\beta_{21}+{\mathrm{bun}}_{i}\beta_{31}+\mathrm{MMSE}1_{i}\beta_{41}\\ & +\mathrm{MMSE}2_{i}\beta_{51}+\mathrm{MMSE}3_{i}\beta_{61}+{\mathrm{hypertension}}_{i}\beta_{71}\\ & +\;\mathrm{fruit}2_{i}\beta_{81}+\mathrm{fruit}3_{i}\beta_{91}+\mathrm{fruit}4_{i}\beta_{101}+t_{\mathrm{ij}}\beta_{111}\\ & +{\tilde{T}}_{i}t_{\mathrm{ij}}\beta_{121}+{\;\gamma }_{i01}+{\;t}_{\mathrm{ij}}\gamma_{i11}+\epsilon_{\mathrm{ij}1} \end{array}$$

(11) $$\begin{array}{ll} \mathrm{Diastolic Blood Pressuree} & =\beta_{02}+\;\mathrm{house}2_{i}\beta_{12}+\mathrm{house}3_{i}\beta_{22}+\mathrm{house}4_{i}\beta_{32}\\ & +\;\mathrm{house}5_{i}\beta_{42}+\;\mathrm{MMSE}1_{i}\beta_{52}+\mathrm{MMSE}2_{i}\beta_{62}\\ & +\;\mathrm{MMSE}3_{i}\beta_{72}+{\mathrm{hypertension}}_{i}\beta_{82}+{\tilde{T}}_{i}\beta_{92}\\ & +{\;t}_{\mathrm{ij}}\beta_{102}+{\tilde{T}}_{i}t_{\mathrm{ij}}\beta_{112}+{\;\gamma }_{i012}+t_{\mathrm{ij}}\gamma_{i12}+\epsilon_{\mathrm{ij}2} \end{array}$$

(12) $$\begin{array}{ll} \mathrm{Total Cholesterol} & =\beta_{03}+{\;\mathrm{sex}}_{i}\beta_{13}+{\mathrm{age}}_{i}\beta_{23}+{\mathrm{prov}}_{i}\beta_{33}+{\mathrm{hct}}_{i}\beta_{43}\\ & +\;\mathrm{house}2_{i}\beta_{53}+\mathrm{house}3_{i}\beta_{63}+\mathrm{house}4_{i}\beta_{73}\\ & +\;\mathrm{house}5_{i}\beta_{83}+\mathrm{MMSE}1_{i}\beta_{93}+\mathrm{MMSE}2_{i}\beta_{103}\;\\ & +\;\mathrm{MMSE}3_{i}\beta_{113}+{\mathrm{glu}}_{i}\beta_{123}+t_{\mathrm{ij}}\beta_{133}+\gamma_{i03}\\ & +{\;t}_{\mathrm{ij}}\gamma_{i13}+\epsilon_{\mathrm{ij}3} \end{array}$$

(13) $$\begin{array}{ll} \mathrm{High-Density Lipoprotein Cholesterol} & =\beta_{04}+{\tilde{T}}_{i}\beta_{14}+{\mathrm{age}}_{i}\beta_{24}+\mathrm{residenc}3_{i}\beta_{34}\\ & +{\;\mathrm{hct}}_{i}\beta_{44}+{\mathrm{hgb}}_{i}\beta_{54}+{\mathrm{glu}}_{i}\beta_{64}+t_{\mathrm{ij}}\beta_{74}+\gamma_{i04}\\ & +{\;t}_{\mathrm{ij}}\gamma_{i14}+\epsilon_{\mathrm{ij}4} \end{array}$$

(14) $$\begin{array}{ll} \mathrm{Red Cell Count} & =\beta_{05}+{\mathrm{prov}}_{i}\beta_{15}+{\mathrm{diabetes}}_{i}\beta_{25}+{\mathrm{hgb}}_{i}\beta_{35}\\ & +\;\mathrm{MMSE}1_{i}\beta_{45}+\mathrm{MMSE}2_{i}\beta_{55}+\mathrm{MMSE}3_{i}\beta_{65}\\ & +\;\mathrm{fruit}2_{i}\beta_{75}+\mathrm{fruit}3_{i}\beta_{85}+\mathrm{fruit}4_{i}\beta_{95}\\ & +{\;t}_{\mathrm{ij}}^{2}\beta_{105}+{\tilde{T}}_{i}\beta_{115}+t_{\mathrm{ij}}\beta_{125}+{\tilde{T}}_{i}t_{\mathrm{ij}}\beta_{135}+\gamma_{i05}\\ & +{\;t}_{\mathrm{ij}}\gamma_{i15}+\epsilon_{\mathrm{ij}5} \end{array}$$

(15) $$\begin{array}{ll} \mathrm{Platelet Count} & =\beta_{06}+{\tilde{T}}_{i}\beta_{16}+{\mathrm{prov}}_{i}\beta_{26}+\mathrm{house}2_{i}\beta_{36}\\ & +\;\mathrm{house}3_{i}\beta_{46}+\mathrm{house}4_{i}\beta_{56}+\mathrm{house}5_{i}\beta_{66}\\ & +\;\mathrm{MMSE}1_{i}\beta_{76}+\mathrm{MMSE}2_{i}\beta_{86}+\mathrm{MMSE}3_{i}\beta_{96}\\ & +{\;t}_{\mathrm{ij}}\beta_{106}+{\tilde{T}}_{i}t_{\mathrm{ij}}\beta_{116}+\gamma_{i06}+t_{\mathrm{ij}}\gamma_{i16}+\epsilon_{\mathrm{ij}6} \end{array}$$

The models constructed by the LTS groups’ longitudinal data were:

(16) $$\begin{array}{ll} \mathrm{Systolic Blood Pressure} & =\beta_{01}^{e}+{\mathrm{bun}}_{i}\beta_{11}^{e}+\mathrm{MMSE}1_{i}\beta_{21}^{e}+\mathrm{MMSE}2_{i}\beta_{31}^{e}\\ & +\;\mathrm{MMSE}3_{i}\beta_{41}^{e}+{\mathrm{hypertension}}_{i}\beta_{51}^{e}+{\;\mathrm{smoke}}_{i}\beta_{61}^{e}\\ & +{\;t}_{\mathrm{ij}}\beta_{71}^{e}+t_{\mathrm{ij}}^{2}\beta_{81}^{e}+{\;\gamma }_{i01}^{e}+t_{\mathrm{ij}}\gamma_{i11}^{e}+\epsilon_{\mathrm{ij}1}^{e} \end{array}$$

(17) $$\begin{array}{ll} \mathrm{Diastolic Blood Pressure} & =\beta_{02}^{e}+{\mathrm{sex}}_{i}\beta_{12}^{e}+{\mathrm{hct}}_{i}\beta_{22}^{e}+{\mathrm{bun}}_{i}\beta_{32}^{e}+{\mathrm{hgb}}_{i}\beta_{42}^{e}\;\\ & +\;\mathrm{house}2_{i}\beta_{52}^{e}+\mathrm{house}3_{i}\beta_{62}^{e}+\mathrm{house}4_{i}\beta_{72}^{e}\\ & +\;\mathrm{house}5_{i}\beta_{82}^{e}+{\mathrm{hypertension}}_{i}\beta_{92}^{e}\;\\ & +{\;t}_{\mathrm{ij}}\beta_{102}^{e}+t_{\mathrm{ij}}^{2}\beta_{112}^{e}+{\;\gamma }_{i02}^{e}+t_{\mathrm{ij}}\gamma_{i12}^{e}\;\\ & +\epsilon_{\mathrm{ij}2}^{e} \end{array}$$

(18) $$\begin{array}{ll} \mathrm{Total Cholesterol} & =\beta_{03}^{e}+{\mathrm{sex}}_{i}\beta_{13}^{e}+{\;\mathrm{age}}_{i}\beta_{23}^{e}+{\;\mathrm{prov}}_{i}\beta_{33}^{e}\\ & +\;\mathrm{residenc}2_{i}\beta_{43}^{e}+\mathrm{residenc}3_{i}\beta_{53}^{e}+{\mathrm{hct}}_{i}\beta_{63}^{e}\\ & +\;\mathrm{house}2_{i}\beta_{73}^{e}+\mathrm{house}3_{i}\beta_{83}^{e}+\mathrm{house}4_{i}\beta_{93}^{e}\\ & +\;\mathrm{house}5_{i}\beta_{103}^{e}+\mathrm{fruit}2_{i}\beta_{113}^{e}+\mathrm{fruit}3_{i}\beta_{123}^{e}\;\\ & +\;\mathrm{fruit}4_{i}\beta_{133}^{e}+t_{\mathrm{ij}}\beta_{143}^{e}+t_{\mathrm{ij}}^{2}\beta_{153}^{e}+\gamma_{i03}^{e}\\ & +{\;t}_{\mathrm{ij}}\gamma_{i13}^{e}+\epsilon_{\mathrm{ij}3}^{e} \end{array}$$

(19) $$\begin{array}{ll} \mathrm{High-Density Lipoprotein Cholesterol} & =\beta_{04}^{e}+{\;\mathrm{smoke}}_{i}\beta_{14}^{e}+{\;\mathrm{glu}}_{i}\beta_{24}^{e}+t_{\mathrm{ij}}\beta_{34}^{e}+{\;\gamma }_{i04}^{e}\\ & +{\;t}_{\mathrm{ij}}\gamma_{i14}^{e}+\epsilon_{\mathrm{ij}4}^{e} \end{array}$$

(20) $$\begin{array}{ll} \mathrm{Red Cell Count} & =\beta_{05}^{e}+{\mathrm{prov}}_{i}\beta_{15}^{e}+{\mathrm{smoke}}_{i}\beta_{25}^{e}+{\mathrm{hgb}}_{i}\beta_{35}^{e}\\ & +\;\mathrm{hypertension}2_{i}\beta_{45}^{e}+t_{\mathrm{ij}}\beta_{55}^{e}+t_{\mathrm{ij}}^{2}\beta_{65}^{e}+\gamma_{i05}^{e}\\ & +{\;t}_{\mathrm{ij}}\gamma_{i15}^{e}+\epsilon_{\mathrm{ij}5}^{e} \end{array}$$

(21) $$\begin{array}{ll} \mathrm{Platelet Count} & =\beta_{06}^{e}+{\mathrm{prov}}_{i}\beta_{16}^{e}+\mathrm{MMSE}1_{i}\beta_{26}^{e}+\mathrm{MMSE}2_{i}\beta_{36}^{e}\\ & +\;\mathrm{MMSE}3_{i}\beta_{46}^{e}+\mathrm{fruit}2_{i}\beta_{56}^{e}+\;\mathrm{fruit}3_{i}\beta_{66}^{e}\;\\ & +\;\mathrm{fruit}4_{i}\beta_{76}^{e}+t_{\mathrm{ij}}\beta_{86}^{e}+\gamma_{i06}^{e}+t_{\mathrm{ij}}\gamma_{i16}^{e}+\epsilon_{\mathrm{ij}6}^{e}.\; \end{array}$$

The goodness of fit of each submodel can be found in the Supplementary Materials (Table S2). We then iterated the fixed and random effect coefficients that were obtained here in the EM algorithm. The final estimated values were used to calculate the probability density function of multivariate normal distribution, $f\left(Y|\tilde{T},Z\right)$, by Equations (7) and (8). The final estimated fixed coefficients and random effect covariance structures can be found in the Supplementary Materials (Tables S3–S5). For Equations (10)–(21), their significance lies in that, while calculating the stroke risk value through the risk factors, they can also describe the relationship between the six main numerical factors and the other 14 remaining factors to conveniently provide personalized prevention suggestions, according to the different risk factors in the applications. For example, we found that MMSE and province (south/north) were most strongly associated with the main numerical factors, because they showed significance (*p*-value < 0.1) in seven and six of the above equations, respectively. In contrast, the effects of diabetes were less influential than in previous studies on this sample of elderly people, as it was only considered in one of the final equations. In terms of the coefficients, it may not be reasonable to directly compare the values of the coefficients between different factors because their interval values are different. For example, the normal range of blood urea nitrogen is 2.1~7.1 mmol/L [28], and erythrocyte hematocrit is commonly in the vicinity of 36.1~50.3% [29]. However, we can still derive the relationship between the remaining factors and the main numerical factors according to the positive and negative coefficients, e.g., hypertension and blood pressure are positively related ($\beta_{71}$, $\beta_{82}$, $\beta_{51}^{e}$, and $\beta_{92}^{e}$). Another interesting finding was the province. Patients in the northern province seem to have higher total cholesterol, red blood cell count, and platelet count, as the coefficients of the northern province were all positive, compared to the default southern province population, representing a positive influence ($\beta_{33}$, $\beta_{15}$, $\beta_{26}$, $\beta_{33}^{e}$, $\beta_{15}^{e}$, and $\beta_{16}^{e}$). According to this, we need to pay more attention to the high value of total cholesterol, red blood cell count, and platelet count for elderly people in the northern province, while the low value of these three factors for elderly people in the southern province needs to be given more attention. Moreover, our study combined the established factors with RF-RFE factors. For RF-RFE selected features, there have been previous studies on the relationship between these factors and strokes, but they have rarely been considered as predictive factors in a model. For instance, the frequencies of housework and fruit consumption were associated with stroke recurrence in hospitalized Chinese patients with a first acute ischemic stroke [30]. MMSE is also a frequently used tool to screen for cognitive impairment in elderly and hospitalized stroke patients [18]. The functional near-infrared spectroscopy (fNIRS) technique monitors variations in hemoglobin during brain activity to study post-stroke recovery [31]. Our research supports that the factors above can also affect biomarker changes and the risk of having a stroke.

### 3.3. Model Performance

This section introduces the performance and results of the model in the following three parts: a prediction accuracy evaluation of the different times of repeated measurements, an individual dynamic stroke risk prediction plot, and comparisons with commonly used classical stroke calculators.

#### 3.3.1. Accuracy Assessment

**Figure 2:**
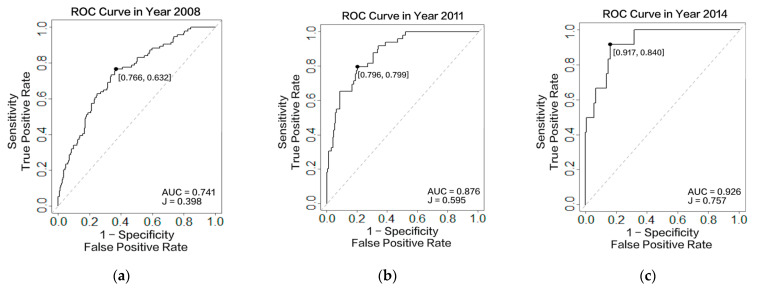
Receiver operating characteristic (ROC) curves for dynamic prediction, with incremental number of measurements. The *x*-axis is the false positive rate, calculated as (1 − specificity) and the *y*-axis is the true positive rate, calculated as sensitivity. Black dots indicate the optimal threshold of ROC curve, with sensitivity and specificity in the bracket. (**a**) ROC curve with one measurement (2008) in 2008; (**b**) ROC curve with two measurements (2008, 2011) in 2011; and (**c**) ROC curve with three measurements (2008, 2011, and 2014) in 2014.

Table 3 and Figure 2 display the results of the prediction accuracy evaluation. We calculated the risk of stroke for each individual over a three-year prediction horizon, based on the model in Section 2.2. These predictions are the result of one, two, and three repeated measurements for everyone in three different years (2008, 2011, and 2014). Table 3 compares the area under the curve (AUC), Youden’s J statistic, sensitivity, specificity, and the threshold of the models when including data from one year, two years, or three years. The following equations calculate these values:

(22) $$\mathrm{Sensitivity}\;(\mathrm{True}\;\mathrm{Positive}\;\mathrm{Rate})=\frac{\mathrm{True}\;\mathrm{Predicted}\;\mathrm{Stroke}}{\mathrm{All}\;\mathrm{Predicted}\;\mathrm{Stroke}}$$

(23) $$\mathrm{Specificity}\;(\mathrm{True}\;\mathrm{Negative}\;\mathrm{Rate})=\frac{\mathrm{True}\;\mathrm{Predicted}\;\mathrm{No}\;\mathrm{Stroke}}{\mathrm{All}\;\mathrm{Predicted}\;\mathrm{No}\;\mathrm{Stroke}}$$

Youden’s J Statistic = Sensitivity + Specificity – 1(24)

After receiving the stroke risk for everyone at each measured time point, we could calculate the sensitivity and specificity by Equations (22) and (23), under different boundary values. The boundary value was used to distinguish between stroke and stroke-free patients at different threshold values, from zero to one. Receiver operating characteristic (ROC) curves were created by plotting the true positive rate against the false positive rate at various threshold settings, presenting the sensitivity or recall as a function of fallout. AUC represents the degree or measure of separability, with a larger value indicating better classification efficiency. Threshold is the optimal threshold for the ROC curve, i.e., the classification value of stroke risk that most accurately distinguishes between the stroke and no-stroke patients. Because a patient’s stroke risk value may change as multiple measurements accumulate, the optimal threshold for the corresponding stroke risk will also change. The sensitivities, specificities, and Youden’s J indices in Table 3 were based on the corresponding optimal thresholds. The results indicated an increase in the AUC value when adding more data acquired during the three measurements, from 0.761 to 0.926. The ROC curves of these three measurements are shown in Figure 2.

To compare the predictive efficiency with other frequently used models, the Cox proportional hazard model with single measurement was also applied to the same sample, as shown in Table 3. The c-indices for the predictions based on 2008, 2011, and 2014 were 0.716, 0.749, and 0.833, respectively. Since the c-index is equal to the AUC value when considering the binary outcome (identified as a stroke or as not in our study) [32], the value can be directly compared. It was found that the AUC values (0.741, 0.876, and 0.926) obtained by the backward joint model and with longitudinal variation consideration, were all higher than the c-indices (0.716, 0.749, and 0.833) obtained by the Cox proportional hazard model. Sensitivity and specificity also increase gradually when we consider more longitudinal data. Youden’s J statistic is also a classic method of summarizing the performance of a diagnostic test, with larger values indicating better prediction accuracy [33]. In our results, Youden’s J statistic increased with more repeated measurements, demonstrating that applying longitudinal data improves prediction accuracy.

Our model refers to the AUC and the Cox proportional hazard model and refers to the c-index. Youden’s J statistic, sensitivity, and specificity are not applicable to the Cox proportional hazard model.

**Figure 3:**
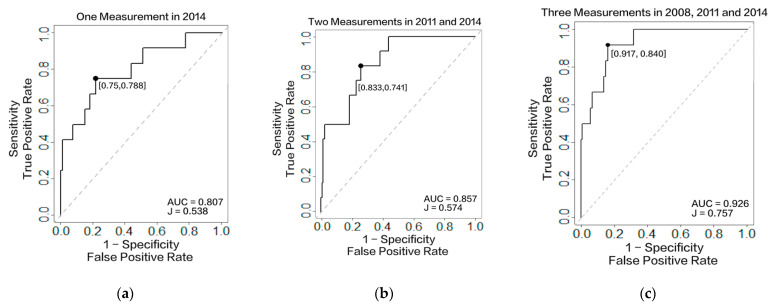
ROC curve for patients who were not identified as having had a stroke in 2014. The *x*-axis is the false positive rate, calculated as (1 – specificity), and the *y*-axis is the true positive rate, calculated as sensitivity. (**a**) ROC curve with one measurement in 2014; (**b**) ROC curve with two measurements in 2011 and 2014; and (**c**) ROC curve with three measurements in 2008, 2011, and 2014.

To better-validate our model, we also compared the prediction accuracy based on the same year of data. Figure 3 presents the 3-year risk of stroke prediction results for patients who were not identified as having had a stroke before 2014. Figure 3a–c are based on one measurement, two measurements, and three measurements, respectively. When comparing the AUC and Youden’s J statistic, both parameters increased with the increasing instances of repeated measurements. More specifically, the AUC value increased from 0.807 to 0.926, and Youden’s J statistic increased from 0.528 to 0.757. This result verifies that considering the longitudinal historical data of features can improve the prediction accuracy. 

#### 3.3.2. Dynamic Stroke Risk Prediction

**Figure 4:**
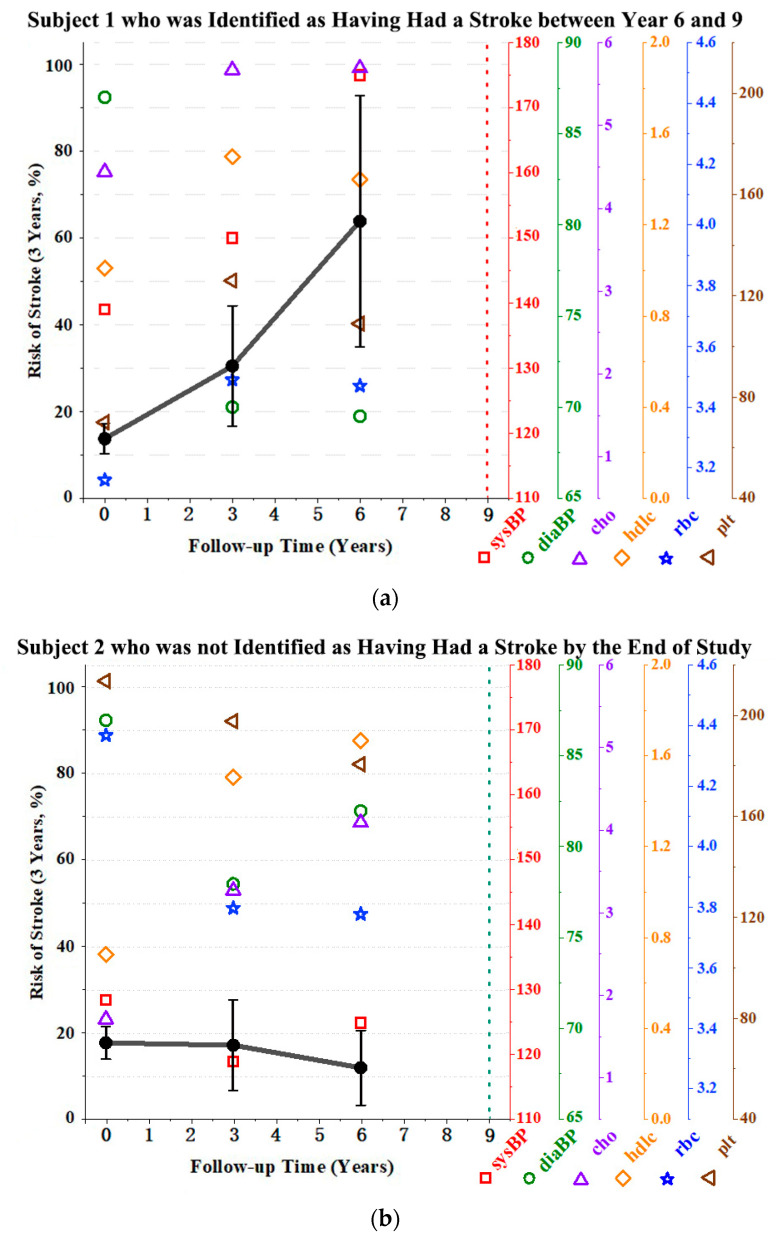
Dynamic risk of stroke prediction for two representative cases. (**a**) Personalized dynamic prediction plot for Subject 1, who was identified as having had stroke between 2014 and 2017; (**b**) personalized dynamic prediction plot for Subject 2, who was not identified as having had a stroke until the end of study (2017). Follow-up years of 0, 3, 6, and 9 equate to 2008, 2011, 2014, and 2017, respectively. The left *y*-axis is the three-year risk of stroke. The right y-axes are values of longitudinal risk factors: sysBP—systolic blood pressure (mmHg); diaBP—diastolic blood pressure (mmHg); cho—total cholesterol (mmol/L); hdlc—high-density lipoprotein cholesterol (mmol/L); rbc—red blood cell count (10^12^/L); and plt—platelet count (10^9^/L). Black dots indicate the three-year risk of stroke at each measurement. Connected plain black lines represent the risk change trend. The vertical interval of each point is the confidence interval calculated by repeated 10-fold cross-validation. The vertical red dotted line is the time point of being diagnosed with a stroke and the vertical green dotted line is the end of the observation time.

Figure 4 shows the longitudinal biomarker trajectories and the dynamic stroke risk predictions of two representative cases from the dataset. Figure 4a presents the dynamic prediction of the risk of stroke for Subject 1, who was identified as having had a stroke after the third measurement (2014–2017). This is an example of someone from the high-risk population. Figure 4b presents the dynamic prediction of Subject 2’s risk of having a stroke, who was not identified as having had a stroke by the end of the study. This subject represents the low-risk group. The black dots indicate the three-year risk of stroke, defined as the risk of developing a first stroke event within three years from the specified time point. According to variations between these two examples, the stroke risk tended to show a noticeable increase over time for the representative subject from the high-risk group (Figure 4a). By contrast, the risk values of the representative subject from the low-risk group were lower and maintained that low value (Figure 4b). Other markers indicated the various factors measured at each time point. It was observed that Subject 1 consistently had higher systolic blood pressure and total cholesterol levels than Subject 2, and these higher physiological parameters were above the normal range (110~150 mmHg for systolic blood pressure and 0~5.18 mmol/L for total cholesterol) [34,35] in the last two measurements. The red blood cell count and platelet count values were lower in Subject 1 compared with Subject 2, and were around the lower boundary of the normal range (3.5~5.5 × 10^12^/L for red cell count and 150~450 × 10^9^/L for platelet count) [36,37]. Other variations in biomarkers showed relatively less differences, but the trends and numerical values still followed the overall variations in the corresponding high-risk and low-risk groups, as shown in Table 1. Overall, based on the personalized dynamic prediction plot, stroke risk and risk factors can be clearly observed in the historical measurements. By adding the newest repeated measurements to obtain the latest results, it is possible to formulate the most appropriate treatment to improve the patient’s lifestyle at any time.

**Figure 5:**
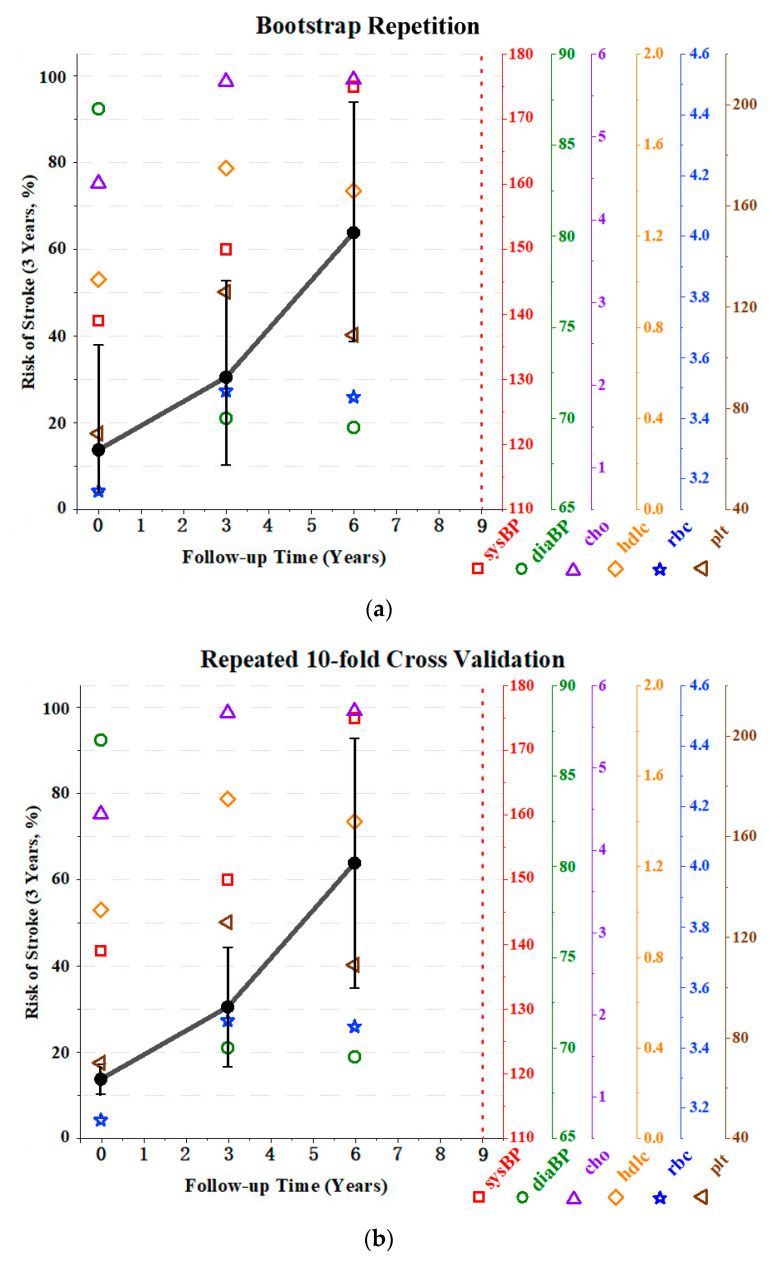
Comparison of the confidence interval using (**a**) bootstrap repetition and (**b**) repeated 10-fold cross-validation on Subject 1. Follow-up years of 0, 3, 6, and 9 equate to 2008, 2011, 2014, and 2017, respectively. The left *y*-axis is the three-year risk of stroke. The right y-axes are values of longitudinal risk factors: sysBP—systolic blood pressure (mmHg); diaBP—diastolic blood pressure (mmHg); cho—total cholesterol (mmol/L); hdlc—high-density lipoprotein cholesterol (mmol/L); rbc—red blood cell count (10^12^/L); and plt—platelet count (10^9^/L). Black dots indicate the three-year risk of stroke at each measurement. Connected plain black lines represent the risk change trend. The vertical interval of each point is the confidence interval calculated by repeated 10-fold cross-validation. The vertical red dotted line is the time point of being identified as having had a stroke.

A comparison of two variance estimation methods based on the same individual was also conducted. Figure 5a is the bootstrap repetition used in the original model, which was proposed by Shen and Li [9], and Figure 5b used repeated ten-fold cross-validation (CV). The total number of simulations was the same. It was shown that the deviations produced by the ten-fold CV were relatively small. However, as the number of measurements considered increased, the resulting confidence interval became wider. Conversely, bootstrap repetitions produced relatively large biases, but the width of the confidence interval became smaller as the number of measurements increased. The difference between the two methods lies in the fact that the idea of bootstrap is completely random replacement sampling, which produces a large bias and a small variance. However, the disadvantage is that it may lead to overfitting and changes in the initial dataset’s distribution. Ten-fold CV enables all data to be involved in training and prediction, and the data distribution is consistent, with a smaller bias and a larger variance. The advantage is that it can avoid overfitting, and the impact of noise is low.

#### 3.3.3. Model Comparisons

**Figure 6:**
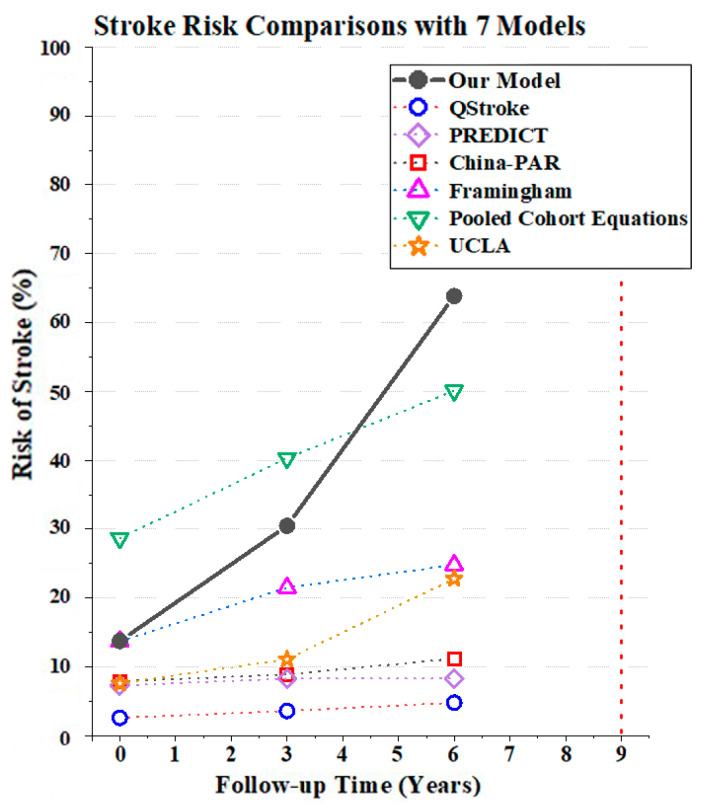
Stroke risk comparisons with seven models based on Subject 1, who was identified as having had a stroke between years 6 and 9. The solid black line and black dots are based on the model used in this paper. Other dotted lines with hollow shapes, in different colors denote the prediction results produced by other stroke risk calculators. Details can be found in the legend and in Table 4.

Stroke risk prediction results vary depending on the databases, risk factors, and algorithms on which they are based. We compared the results of six commonly used stroke risk calculators with our model. Unlike our model, the calculated stroke risks from other models were only based on single-time measured stroke risk factors. The comparison was based on three measurements from Subject 1, who was identified as having had a stroke between years six and nine. All the results are presented in Figure 6 and Table 4. The most apparent abnormal biomarkers for this individual were systolic blood pressure and total cholesterol. In the first measurement, the subject had 139 mmHg systolic blood pressure and 4.44 mmol/L total cholesterol. These values were high but still within the normal range (110~150 mmHg for systolic blood pressure and 0~5.18 mmol/L for total cholesterol) [34,35]. Therefore, it was acceptable for her to be predicted as being at low risk by our model, and no active reaction was needed. However, in the second measurement, these two biomarkers increased to 150 mmHg and 5.67 mmol/L, over the normal range, and continued rising in the third measurement. This led to a prediction result of high-risk in the subject’s subsequent two predictions by our model. If they had some prevention or treatment strategy, then their stroke risk value and level might have decreased in the third prediction.

From the individual prediction result’s perspective, in all models, the numerical values of stroke risk were increased, which also verifies the accuracy of our model. In addition, the numerical results calculated by our model rose more significantly, so it can serve as a better warning for high-risk group patients. According to the results presented, some models provide a reference risk level based on their models. Our model also classifies the prediction results into high-risk and low-risk groups. The boundary value is the risk classification value corresponding to the optimal threshold of the ROC curve, representing the classification value of the stroke risk that most accurately distinguishes between high- and low-risk patients. These values can be found in Table 3, in the “Threshold” column. The China-PAR model and the Framingham study calculated similar changes in risk levels to ours. Although UCLA showed risk value increases, it consistently classified the results as low-risk. QStroke, PREDICT, and pooled cohort equations calculated the risk values without defined risk levels, e.g., a result of 2.6% for QStroke represented that 2.6 out of 100 patients with the same risk factors were likely to have a stroke in the next ten years. However, patients cannot understand whether the value indicates low- or high-risk without a level classification. For example, 11.2% is classified as high-risk in the China-PAR model, but as low-risk in our model. Therefore, this again proves the importance of the corresponding reference value of risk stratification. 

In terms of the prediction horizon, most existing models consider ten years. On the one hand, if a patient is predicted to be at high-risk, warning and preventive measures could be suggested at an early stage. However, on the other hand, because the 10-year prediction horizon is too broad, it is difficult to determine the exact stroke onset. Moreover, for the elderly, a premature warning can easily cause psychological panic. Only the PREDICT model used a five-year prediction horizon and QStroke can calculate the three-year risk of stroke. Therefore, the three-year prediction range provided by our model can give patients narrower and more precise prediction information.

From the perspective of the overall model, we listed the C-index and 95% CI for all available models, and some have sex-specific equations. All the information can be found in Table 5. We found that, although the prediction efficiency of our model with a single measurement was lower than some models, the accuracy rose and became the highest when considering repeated measurements at 0.926. In addition, we applied our dataset to two available open models, the China-PAR model and the Framingham study. All predictions were based on a single measurement at each measured time point and the corresponding C-index can be found in Table 6. Both models have unsatisfactory prediction results on our dataset, with C-indices around 0.55. One reason might be that the target population in both datasets focused mainly on middle-aged people from 30 to 74, whereas our dataset focused on those aged 62 to 105. This, again, suggests that it is necessary to construct specific models for the elderly in China. Moreover, for these older people, the prediction results tended to be higher for all high- and low-risk patients. However, in our model, the specificity, which reflects the prediction accuracy of low-risk patients, increased significantly with the increment of repeated measurements (from 0.632 to 0.840). This indicates that our prediction model is more friendly to low-risk patients than the other two models.

## 4. Discussion

Stroke epidemiology shows that the morbidity of strokes in China increases with age. As the life expectancy lengthens and the proportion of older people increases, strokes become more severe in the elderly [42]. Therefore, it is a crucial challenge for China, in the future, to solve the problem of how to efficiently deal with stroke prevention and management, and to achieve the reasonable allocation of medical treatment. 

We compared our results with those from prior publications on stroke risk prediction, based on the Chinese population. For example, Wu and Fang (2020) chose the same dataset as us, using an SVM and decision tree, but only selected 2011 and 2014 for the baseline and the predicted results. The highest AUC of their prediction model was 0.72, with 0.75 sensitivity and 0.69 specificity [43]. Another study we considered was the China-PAR project, which used the simple Cox proportional hazard model and received a c-index of 0.794 for males and 0.811 for females [26]. As for longitudinal data, the most used model is the joint model. For example, Kang et al. used the joint model to discover the conversion to Alzheimer’s disease [44]. In this paper, we first used the backward joint model (BJM), a new algorithm that has recently been proposed, so there are limited examples of its use in recent cases. Compared with other joint models, the BJM has the advantage of resolving the issue that ordinary joint models must contain time-to-event data, and it supports the consideration of censored data for model construction. Furthermore, it can consider multiple longitudinal factors with simple calculations, since the BJM contains only a one-dimensional integral in the censored time domain in E-step, and a closed-form solution in M-step for the EM algorithm. In comparison with machine-learning algorithms, although fewer factors are considered, the relationships between the risk factors and stroke risk values are more apparent. Therefore, clinicians can provide personalized prevention suggestions based on this information. Meanwhile, compared with the Cox proportional hazard model, the BJM can consider more predictive variables and variations to improve the prediction accuracy. Second, our results with longitudinal data consideration achieved higher AUC (0.741,0.876, 0.926), sensitivity (0.766, 0.796, 0.917), and specificity values (0.632, 0.799, 0.840), showing better predictive accuracy. This is the first time, as far as we are aware, that health measurement longitudinal data have been used in stroke risk prediction and as a new direction for using EHRs to run predictions. Moreover, the model can also reflect variations in patients’ stroke risk value and risk factors in real time.

In terms of the dataset selection, the CLHLS-HF dataset was a prospective cohort study of elderly people in China, which has been widely used in the medical field. For example, it has been used to explore the relationship between the time of first smoking and the prevalence of chronic respiratory diseases [45], and the association between socioeconomic status and health in elderly people with diabetes [46]. Because it also collected information related to the risk of strokes, we believed it would be valuable to use for stroke risk prediction. In terms of the measurement period, we believed six years was an appropriate time between the first and last observations, as the research conducted by Zhao et al. used a similar observation window: seven years [10]. In our model, the predictive variables have high accessibility, meaning no costly or time-consuming examinations are needed. Thus, this prediction model can be included in annual health examinations or be applied to the preliminary screening of large-scale, high-risk populations, and provide guidance for stroke prevention and management in the elderly.

There were also some limitations to the study. First, most of the information was collected through questionnaires, by self-reporting, which led to subject bias. Moreover, the implemented dataset was not targeted at stroke research, so the final sample that could be considered in the research was limited. To construct a model with higher accuracy and wider applicability, it is crucial to obtain a larger dataset (e.g., >1000), which targets cardiovascular disease in the future. It would also be meaningful to differentiate the final status more specifically, e.g., to distinguish between ischemic and hemorrhagic strokes, since their risk factors have been proven to be different [47]. In terms of data collection, CLHLS-HF was measured every three years. Some longitudinal variations have been observed, but more frequent continual monitoring is believed to provide higher accuracy and more timely results. In terms of future applications, it is expected to be used for EHRs or annual health examinations. Moreover, it can be combined with wearable devices to obtain accurate physiological data in real time [48]. Finally, since there was only one dataset in this research, the generalization ability of the model still needs to be tested. Therefore, external validation by other datasets is necessary for future research.

In terms of its future applications, this model can be implemented to produce a user interface or application. When patients provide their information on a form, the model determines the risk values and the corresponding risk categories. Participants are encouraged to input their health records as frequently as possible to increase the prediction accuracy. When inputting multiple records, an individual dynamic risk prediction plot would be produced to reflect the variations in the biomarkers and stroke risk over time. Clinicians will be invited to optimize the prediction model to provide personalized care. Because this model is flexible, it can be easily updated with new measurements.

## 5. Conclusions

This paper demonstrates that applying the backward joint model to longitudinal data attains high-precision predictions of the risk of strokes. Our model achieves 0.926 accuracy when considering three longitudinal measurements and has a higher prediction accuracy than other conventional risk scales. In addition, 10 out of the 20 risk factors, which are not commonly applied in other risk scales, were found to be useful for stroke risk prediction. These predictors include red cell count, platelet count, erythrocyte hematocrit, blood urea nitrogen, hemoglobin, glucose, frequency of doing housework, frequency of fruit consumption, mini-mental state examination, and hypertension history. Regarding variance, 10-fold cross-validation was chosen to avoid overfitting and to involve all data in the model construction and validation. In terms of outcomes, dynamic prediction allows for better monitoring of the value of stroke risk and variations in physical health, and in assisting clinicians in formulating corresponding treatments and prevention strategies. It is promising that the proposed model could be combined with health examination data or electronic health records to further improve the prediction precision. We believe this is a new advancement in real-time prediction. In the future, it is expected that better quality, more frequently assessed, and larger sample sizes will be available, combined with multimodal data, such as from wearable devices, to better monitor the variations in risk factors for strokes. The goal of using longitudinal data for dynamic prediction is timely stroke risk monitoring and the rational allocation of medical resources. 

## Figures and Tables

**Table 1:** Average and standard deviation of six main numerical risk factors at each measurement for stroke and no-stroke individuals.

	Stroke		No Stroke	
	Average	SD	Average	SD
2008 (155/162)				
Systolic Blood Pressure (mmHg) *	143	21.75	140.5	18.31
Diastolic Blood Pressure (mmHg) *	79.5	11.60	79	9.98
Total Cholesterol (mmol/L) *	3.71	1.34	3.22	1.26
High-Density Lipoprotein Cholesterol (mmol/L) *	1.16	0.34	1.06	0.35
Red Cell Count (10^12^/L)	5.68	2.73	5.95	2.76
Platelet Count (10^9^/L)	248.48	156.93	250.97	138.59
2011 (61/162)				
Systolic Blood Pressure (mmHg) *	134.5	16.85	138	19.63
Diastolic Blood Pressure (mmHg) *	83.5	11.38	83	11.20
Total Cholesterol (mmol/L) *	4.36	1.00	4.14	0.92
High-Density Lipoprotein Cholesterol (mmol/L) *	1.35	0.40	1.22	0.34
Red Cell Count (10^12^/L)	4.36	1.54	4.91	1.65
Platelet Count (10^9^/L)	167.79	83.78	219.28	97.48
2014 (12/162)				
Systolic Blood Pressure (mmHg) *	138	26.13	143	22.35
Diastolic Blood Pressure (mmHg) *	75.5	9.46	81.5	12.61
Total Cholesterol (mmol/L) *	4.99	1.11	4.70	0.99
High-Density Lipoprotein Cholesterol (mmol/L) *	1.41	0.50	1.367	0.39
Red Cell Count (10^12^/L)	4.19	0.86	4.34	0.80
Platelet Count (10^9^/L)	152.78	66.31	195.50	60.26

Factors with * are established factors. SD—standard deviation. 2008, 2011, and 2014 are the years longitudinal data were measured. The information in brackets after the years are the number of stroke individuals/number of no-stroke individuals.

**Table 2:** Characteristics of remaining risk factors at each measurement for stroke and no-stroke individuals.

	Abbreviations in Equations (10)–(21)	Stroke	No stroke
		Mean (SD)	Mean (SD)
2008 (155/162)			
Sex *	sex	**Female:** 77, **Male:** 78	**Female:** 73, **Male:** 89
Age *	age	79.2 (11.96)	76.3 (10.41)
Provenience *	prov	**South:** 97, **North:** 58	**South:** 98, **North:** 64
Residence Location *	residenc	**City:** 0, **Town:** 23, **Rural:** 132	**City:** 5, **Town:** 30, **Rural:** 127
Diabetes History *	diabetes	**No:** 149, **Yes:** 6	**No:** 159, **Yes:** 3
Smoke (number per day) *	smoke	2.8 (5.60)	3.7 (10.41)
**Erythrocyte Hematocrit (%)**	hct	45.41 (15.48)	45.60 (12.99)
**Blood Urea Nitrogen (mmol/L)**	bun	6.19 (1.82)	6.20 (1.79)
**Hemoglobin (g/L)**	hgb	135.2 (23.25)	140 (22.34)
**Housework**	house	**1:** 90, **2:** 17, **3:** 3, **4:** 10, **5:** 35	**1:** 120, **2:** 8, **3:** 10, **4:** 3, **5:** 21
**MMSE**	MMSE	**0:** 70, **1:** 42, **2:** 25, **3:** 18	**0:** 92, **1:** 50, **2:** 12, **3:** 8
**Hypertension History**	hypertension	**No:** 128, **Yes:** 27	**No:** 150, **Yes:** 12
**Fruit Consumption**	fruit	**1:** 7, **2:** 35, **3:** 67, **4:** 46	**1:** 16, **2:** 59, **3:** 66, **4:** 21
**Glucose (mmol/L)**	glu	5.65 (2.32)	5.26 (1.80)
2011 (61/162)			
Sex *	sex	**Female:** 45, **Male:** 16	**Female:** 73, **Male:** 89
Age *	age	80.4 (11)	79.3 (10.41)
Provenience *	prov	**South:** 45, **North:** 16	**South:** 98, **North:** 64
Residence Location *	residenc	**City:** 0, **Town:** 7, **Rural:** 54	**City:** 5, **Town:** 30, **Rural:** 127
Diabetes History *	diabetes	**No:** 58, **Yes:** 3	**No:** 152, **Yes:** 10
Smoke (number per day) *	smoke	1.48 (4.41)	3.01 (6.77)
**Erythrocyte Hematocrit (%)**	hct	40.09 (7.62)	42.07 (10.92)
**Blood Urea Nitrogen (mmol/L)**	bun	6.86 (1.71)	6.48 (1.70)
**Hemoglobin (g/L)**	hgb	133 (21.72)	136.2 (25.64)
**Housework**	house	**1:** 32, **2:** 4, **3:** 2, **4:** 5, **5:** 18	**1:** 105, **2:** 19, **3:** 3, **4:** 6, **5:** 29
**MMSE**	MMSE	**0:** 43, **1:** 7, **2:** 5, **3:** 6	**0:** 114, **1:** 30, **2:** 12, **3:** 6
**Hypertension History**	hypertension	**No:** 37, **Yes:** 24	**No:** 112, **Yes:** 50
**Fruit Consumption**	fruit	**1:** 5, **2:** 12, **3:** 31, **4:** 13	**1:** 9, **2:** 44, **3:** 73, **4:** 36
**Glucose (mmol/L)**	glu	4.32 (3.78)	4.40 (1.56)
2014 (12/162)			
Sex *	sex	**Female:** 7, **Male:** 5	**Female:** 73, **Male:** 89
Age *	age	80.5 (6.87)	82.3 (10.41)
Provenience *	prov	**South:** 9, **North:** 3	**South:** 98, **North:** 64
Residence Location *	residenc	**City:** 0, **Town:** 1, **Rural:** 11	**City:** 5, **Town:** 30, **Rural:** 127
Diabetes History *	diabetes	**No:** 10, **Yes:** 2	**No:** 136, **Yes:** 26
Smoke (number per day) *	smoke	0.83 (2.89)	2.82 (6.91)
**Erythrocyte Hematocrit (%)**	hct	39.7 (8.31)	40.18 (6.68)
**Blood Urea Nitrogen (mmol/L)**	bun	6.48 (1.94)	6.15 (1.67)
**Hemoglobin (g/L)**	hgb	130.8 (25.56)	132.3 (19.20)
**Housework**	house	**1:** 11, **2:** 0, **3:** 0, **4:** 0, **5:** 1	**1:** 100, **2:** 13, **3:** 3, **4:** 4, **5:** 42
**MMSE**	MMSE	**0:** 9, **1:** 2, **2:** 1, **3:** 0	**0:** 115, **1:** 25, **2:** 11, **3:** 11
**Hypertension History**	hypertension	**No:** 8, **Yes:** 4	**No:** 92, **Yes:** 70
**Fruit Consumption**	fruit	**1:** 2, **2:** 5, **3:** 5, **4:** 0	**1:** 12, **2:** 56, **3:** 65, **4:** 29
**Glucose (mmol/L)**	glu	5.094 (0.78)	5.34 (1.46)

Factors with * are established factors. SD—standard deviation. 2008, 2011, and 2014 are the years longitudinal data were measured. The information in brackets after the years are the number of stroke individuals/number of no-stroke individuals). Housework (frequency of doing housework): 1 = almost every day; 2 = at least once a week; 3 = not every week, but at least once a month; 4 = not every month, but sometimes; and 5 = never. MMSE (mini-mental state examination): 0 = normal; 1 = mild; 2 = medium; and 3 = severe. Fruit consumption (frequency of fruit consumption): 1 = almost every day; 2 = quite often; 3 = occasionally; and 4 = rarely or never.

**Table 3:** Prediction accuracy of the proposed model with different numbers of measurements.

		AUC/ C-Index	Youden’s J Statistic	Sensitivity	Specificity	Threshold
**Our Model**	**One Measurement Obtained in 2008**	0.741	0.398	0.766	0.632	0.242
	**Two Measurements Obtained in 2008 and 2011**	0.876	0.595	0.796	0.799	0.182
	**Three Measurements Obtained in 2008, 2011, and 2014**	0.926	0.757	0.917	0.840	0.107
**Cox Proportional Hazard Model**	**One Measurement Obtained in 2008**	0.716	NA	NA	NA	NA
	**One Measurement Obtained in 2011**	0.749	NA	NA	NA	NA
	**One Measurement Obtained in 2014**	0.833	NA	NA	NA	NA

**Table 4:** Stroke risk comparison information, applying seven models to the case of Subject 1. The stroke risk levels are also shown when available.

Models	Risk of Stroke			Prediction Horizon
	2008	2011	2014	
**Our Model**	13.75% Low-risk	30.43% High-risk	63.87% High-risk	3 Years
**QStroke [38]**	2.60%	3.60%	4.80%	3 Years
**PREDICT [39]**	7.30%	8.30%	8.30%	5 Years
**China-PAR [26]**	7.90% Medium-risk	8.90% Medium-risk	11.2% High-risk	10 Years
**Framingham [40]**	13.70% Medium-risk	21.5% High-risk	24.8% High-risk	10 Years
**Pooled Cohort Equations [25]**	28.70%	40.30%	50.10%	10 Years
**UCLA [41]**	7.60% Low-risk	11.10% Low-risk	22.80% Low-risk	10 Years

**Table 5:** Table of AUC/C-index values of five available models and our model. The 95% CI of C-index was also shown when available.

Model		C-Index/AUC	95% CI
**Our Model**		0.741–0.926	
**QStroke**	Male	0.71	[0.69,0.73]
	Female	0.65	[0.62,0.67]
**PREDICT**		0.73	[0.72–0.73]
**China-PAR**	Male	0.794	[0.775,0.814]
	Female	0.811	[0.787,0.835]
**Framingham**	Male	0.763	[0.746,0.790]
	Female	0.793	[0.772,0.814]
**Pooled Cohort Equation**		0.713–0.814	

**Table 6:** Table of C-index results based on China-PAR model and Framingham study, implemented on our dataset (number of stroke individual/number of no stroke individual).

Year	Status	C-Index	
		China-PAR Model	Framingham Study
**2008**	**(155/162)**	0.522	0.552
**2011**	**(61/162)**	0.514	0.538
**2014**	**(12/162)**	0.617	0.584

## Data Availability

The Chinese Longitudinal Healthy Longevity and Happy Family Study (CLHLS-HF) dataset used in this study was collected by the Centre for Healthy Aging and Development Studies of the National School of Development, at Peking University. The dataset is open access for personal academic or policy research activities. It can be accessed here: https://opendata.pku.edu.cn/dataverse/CHADS (accessed on 22 November 2021).

## Supplementary Materials

The following supporting information can be downloaded at: https://www.mdpi.com/article/10.3390/healthcare10112134/s1. Table S1: the *p*-value table for 12 numerical features to distinguish the main numerical and remaining factors; Table S2: the goodness of fit of the mixed linear-effects model from Equations (10) to (21); Table S3: the fixed effect coefficients β, obtained from Equations (10) to (21) and estimated after *m*th iteration of the EM algorithm; Table S4: the random effect covariance structure table Ω for the stroke individual, with ${\tilde{T}}_{i}\;\leq \;\tau$ obtained from Equations (10) to (15) and estimated after *m*th iteration of the EM algorithm; Table S5: the random effect covariance structure table $\Omega^{e}$ for the LTS individual with ${\tilde{T}}_{i}>\tau$, obtained from Equations (16) to (21) and estimated after *m*th iteration of the EM algorithm.
