# Supplementary material for: Longitudinal Data to Enhance Dynamic Stroke Risk Prediction
Source: Healthcare (Basel). 2022 Oct 27;10(11):2134. doi: 10.3390/healthcare10112134 (PMC9691140; doi:10.3390/healthcare10112134)
Supplement: Supplementary file 1 [file healthcare-10-02134-s001.zip › healthcare-1950477-supplementary.pdf]

Supplementary Materials

Longitudinal Data to Enhance Dynamic Stroke Risk Prediction

Wenyao Zheng, Yun-Hsuan Chen, and Mohamad Sawan

Table S1. The p-value table for 12 numerical features to distinguish the main numerical and remaining factors. All the p-values were calculated between the stroke and no stroke groups by Welch's t-test. The second to the fourth columns are based on single-time measurement data. The fifth to the sixth columns are based on the difference between two measurements. The * represents the p-value <0.05, indicating that this feature has significant differences between two groups. (Number of stroke individual/Number of no stroke individual)

Features	P-values				
	2008 (94/223)	2011 (49/174)	2014 (12/162)	Difference between 2008-2011 (61/162)	Difference between 2011-2014 (12/162)
**Systolic Blood Pressure**	0.0489*	0.0438*	0.5671	0.0411*	0.4190
**Diastolic Blood Pressure**	0.9836	0.8408	0.0635	0.8922	0.0136*
**Total Cholesterol**	0.0288*	0.3171	0.3845	0.1564	0.7766
**High-density Lipoprotein Total Cholesterol**	0.0072*	0.0372*	0.7919	0.1531	0.7060
**Red Blood Cell Count**	0.1070	0.0956	0.5697	0.0449*	0.0376*
**Platelet Count**	0.0211*	0.0001*	0.0504	0.7890	0.5694
**Age**	0.0106*	0.3064	0.4139	NA	NA
**Smoke**	0.3397	0.1460	0.0582	0.3065	0.6608
**Erythrocyte Hematocrit**	0.3816	0.0964	0.8487	0.8136	0.4302
**Blood Urea Nitrogen**	0.9450	0.0744	0.5826	0.1128	0.4100
**Hemoglobin**	0.3242	0.1660	0.8517	0.0798	0.1016
**Glucose**	0.7586	0.6968	0.3416	0.1461	0.5186

Table S2. The goodness of fit of the mixed linear-effects model from Equations (10) to (21). All values are measured by R^2. In general, $R^2 > 0.5$ is acceptable.

	Model for $\widetilde{T}_i \le \tau$	Model for $\widetilde{T}_i > \tau$
**Systolic Blood Pressure**	0.553	0.743
**Diastolic Blood Pressure**	0.567	0.680
**Total Cholesterol**	0.888	0.594
**High-density Lipoprotein Cholesterol**	0.707	0.764
**Platelet Count**	0.926	0.691
**Red Blood Cell Count**	0.893	0.675

Table S3. The fixed effect coefficients $\boldsymbol{\beta}$ obtained from Equations (10) to (21) and were estimated after mth iteration of the EM algorithm. It was then used in the probability density function of multivariate normal

distribution, $f(Y|\tilde{T}, Z)$, by Equation (7) and (8). Factors corresponding to $\boldsymbol{\beta}$ can be found in Equations (10) to (21). Model for $\tilde{T}_i \leq \tau$ are used for stroke individuals, and Model for $\tilde{T}_i > \tau$ are used for non-stroke individuals.

	**Model for $\tilde{T}_i \leq \tau$**		**Model for $\tilde{T}_i > \tau$**	
**Systolic Blood Pressure**	β_{01}	184.042	$\beta_{01}{}^{e}$	147.451
	β_{11}	-4.197	$\beta_{11}{}^{e}$	-0.949
	β_{21}	0.210	$\beta_{21}{}^{e}$	0.416
	β_{31}	-1.521	$\beta_{31}{}^{e}$	10.494
	β_{41}	1.022	$\beta_{41}{}^{e}$	3.072
	β_{51}	-0.804	$\beta_{51}{}^{e}$	19.315
	β_{61}	-10.318	$\beta_{61}{}^{e}$	-0.288
	β_{71}	13.362	$\beta_{71}{}^{e}$	-2.829
	β_{81}	-13.264	$\beta_{81}{}^{e}$	0.399
	β_{91}	-9.095		
	β_{101}	-6.533		
	β_{111}	-11.685		
	β_{121}	1.273		
**Diastolic Blood Pressure**	β_{02}	74.031	$\beta_{02}{}^{e}$	72.442
	β_{12}	-2.188	$\beta_{12}{}^{e}$	-2.944
	β_{22}	-0.889	$\beta_{22}{}^{e}$	-0.066
	β_{32}	0.436	$\beta_{32}{}^{e}$	-0.897
	β_{42}	-3.958	$\beta_{42}{}^{e}$	0.084
	β_{52}	3.210	$\beta_{52}{}^{e}$	0.903
	β_{62}	-0.573	$\beta_{62}{}^{e}$	5.127
	β_{72}	1.556	$\beta_{72}{}^{e}$	2.688
	β_{82}	6.514	$\beta_{82}{}^{e}$	-1.611
	β_{92}	0.531	$\beta_{92}{}^{e}$	5.239
	β_{102}	4.050	$\beta_{102}{}^{e}$	2.627
	β_{112}	-0.452	$\beta_{112}{}^{e}$	-0.278
**Total Cholesterol**	β_{03}	0.831	$\beta_{03}{}^{e}$	3.017
	β_{13}	-0.722	$\beta_{13}{}^{e}$	-0.417
	β_{23}	0.026	$\beta_{23}{}^{e}$	-0.010
	β_{33}	0.698	$\beta_{33}{}^{e}$	0.407
	β_{43}	0.026	$\beta_{43}{}^{e}$	0.845
	β_{53}	-0.349	$\beta_{53}{}^{e}$	0.826
	β_{63}	0.038	$\beta_{63}{}^{e}$	0.013
	β_{73}	0.683	$\beta_{73}{}^{e}$	0.454
	β_{83}	0.142	$\beta_{83}{}^{e}$	-0.088

	β_{93}	-0.293	$\beta_{93}{}^{e}$	1.248
	β_{103}	-0.743	$\beta_{103}{}^{e}$	0.089
	β_{113}	-0.488	$\beta_{113}{}^{e}$	-0.454
	β_{123}	0.089	$\beta_{123}{}^{e}$	-0.345
	β_{133}	0.215	$\beta_{133}{}^{e}$	-0.140
			$\beta_{143}{}^{e}$	0.413
			$\beta_{153}{}^{e}$	-0.021
**High-density Lipoprotein Cholesterol**	β_{04}	0.668	$\beta_{04}{}^{e}$	1.212
	β_{14}	-0.024	$\beta_{14}{}^{e}$	-0.006
	β_{24}	0.006	$\beta_{24}{}^{e}$	-0.035
	β_{34}	-0.157	$\beta_{34}{}^{e}$	0.051
	β_{44}	0.008		
	β_{54}	0.002		
	β_{64}	-0.034		
	β_{74}	0.065		
**Red Blood Cell Count**	β_{05}	-0.430	$\beta_{05}{}^{e}$	3.526
	β_{15}	4.326	$\beta_{15}{}^{e}$	1.159
	β_{25}	-1.080	$\beta_{25}{}^{e}$	0.026
	β_{35}	0.025	$\beta_{35}{}^{e}$	0.016
	β_{45}	0.216	$\beta_{45}{}^{e}$	0.571
	β_{55}	0.252	$\beta_{55}{}^{e}$	-0.480
	β_{65}	0.948	$\beta_{65}{}^{e}$	0.027
	β_{75}	1.209		
	β_{85}	1.157		
	β_{95}	1.313		
	β_{105}	0.044		
	β_{115}	-0.002		
	β_{125}	0.264		
	β_{135}	-0.084		
**Platelet Count**	β_{06}	245.591	$\beta_{06}{}^{e}$	288.066
	β_{16}	-12.999	$\beta_{16}{}^{e}$	23.239
	β_{26}	179.506	$\beta_{26}{}^{e}$	23.816
	β_{36}	6.994	$\beta_{36}{}^{e}$	4.666
	β_{46}	19.717	$\beta_{46}{}^{e}$	1.793
	β_{56}	17.908	$\beta_{56}{}^{e}$	-51.369
	β_{66}	49.036	$\beta_{66}{}^{e}$	-52.651
	β_{76}	33.338	$\beta_{76}{}^{e}$	-46.077

β_{86}	28.976	${\beta_{86}}^{e}$	-9.245
β_{96}	8.155		
β_{106}	-44.896		
β_{116}	4.343		

Table S4. The random effect covariance structure table $\boldsymbol{\Omega}$ for the stroke individual with $\tilde{T}_i \leq \tau$ obtained from Equations (10) to (15) and were estimated after mth iteration of the EM algorithm. It was then used in the probability density function of multivariate normal distribution, $f(Y|\tilde{T},Z)$, in Equation (7). Referring to the Row and Column name, we can receive specific $\boldsymbol{\Omega}_{gg}$ and $\boldsymbol{\Omega}_{g_1g_2}(g_1 \neq g_2)$, where g is used to index the main numerical factors.

	$\boldsymbol{\Omega_1}$		$\boldsymbol{\Omega_2}$		$\boldsymbol{\Omega_3}$		$\boldsymbol{\Omega_4}$		$\boldsymbol{\Omega_5}$		$\boldsymbol{\Omega_6}$	
$\boldsymbol{\Omega_1}$	487.634	-135.073	84.854	-9.987	4.026	-0.962	0.004	-0.238	-1.710	-1.847	51.453	-21.191
	-135.073	42.962	-15.875	3.679	-0.248	0.230	0.012	0.124	0.747	0.545	11.989	9.332
$\boldsymbol{\Omega_2}$	84.854	-15.875	29.146	-0.693	1.045	-0.138	-0.119	0.045	-0.453	-0.599	-92.336	21.514
	-9.987	3.679	-0.694	0.504	0.346	-0.039	0.023	0.016	-0.057	-0.034	6.370	-1.376
$\boldsymbol{\Omega_3}$	4.026	-0.248	1.045	0.346	1.574	-0.266	0.132	-0.017	-0.002	0.001	5.947	0.864
	-0.962	0.230	-0.138	-0.039	-0.266	0.073	-0.031	0.004	-0.018	0.004	0.495	0.223
$\boldsymbol{\Omega_4}$	0.004	0.012	-0.119	0.023	0.132	-0.031	0.042	0.002	0.042	0.020	3.137	-0.508
	-0.238	0.124	0.045	0.016	-0.017	0.004	0.002	0.003	-0.007	-0.007	1.604	-0.397
$\boldsymbol{\Omega_5}$	-1.710	0.747	-0.453	-0.057	-0.002	-0.018	0.042	-0.007	0.476	0.242	28.349	-0.890
	-1.847	0.546	-0.599	-0.034	0.001	0.004	0.020	-0.007	0.242	0.153	10.429	0.524
$\boldsymbol{\Omega_6}$	51.453	11.989	-92.336	6.370	5.947	0.495	3.137	1.604	28.349	10.429	11326.44	-1921.59
	-21.191	9.332	21.514	-1.376	0.864	0.2223	-0.508	-0.397	-0.890	0.524	-1921.59	421.052

Table S5. The random effect covariance structure table $\boldsymbol{\Omega}^{e}$ for the LTS individual with $\tilde{T}_i > \tau$ obtained from Equations (16) to (21) and were estimated after mth iteration of the EM algorithm. It was then used in the probability density function of multivariate normal distribution, $f(Y|\tilde{T},Z)$, in Equation (8). Referring to the Row and Column name, we can receive $\boldsymbol{\Omega}^{e}_{gg}$ and $\boldsymbol{\Omega}^{e}_{g_1g_2}$ $(g_1 \neq g_2)$, where g is used to index the main numerical factors.

	$\boldsymbol{\Omega^e_1}$		$\boldsymbol{\Omega^e_2}$		$\boldsymbol{\Omega^e_3}$		$\boldsymbol{\Omega^e_4}$		$\boldsymbol{\Omega^e_5}$		$\boldsymbol{\Omega^e_6}$	
$\boldsymbol{\Omega^e_1}$	128.704	-23.288	51.531	-9.839	-0.344	-0.307	0.528	-0.072	2.087	-0.410	155.399	-24.672
	-23.288	8.583	-6.534	3.295	0.046	0.016	-0.251	0.015	-1.015	0.190	-78.526	11.218
$\boldsymbol{\Omega^e_2}$	51.531	-6.534	36.490	-5.111	1.677	-0.202	0.907	-0.057	-0.792	0.170	-27.519	6.736
	-9.839	3.295	-5.111	1.724	-0.253	0.023	-0.209	0.012	0.202	-0.040	7.911	-1.347
$\boldsymbol{\Omega^e_3}$	-0.344	0.046	1.677	-0.253	0.718	-0.098	0.158	-0.022	0.243	-0.063	20.548	-3.676
	-0.307	0.016	-0.202	0.023	-0.098	0.022	-0.013	0.003	-0.079	0.020	-3.308	0.912
$\boldsymbol{\Omega^e_4}$	0.528	-0.251	0.907	-0.209	0.158	-0.013	0.079	-0.004	-0.048	0.007	-0.060	0.103
	-0.072	0.015	-0.057	0.012	-0.022	0.003	-0.004	0.002	-0.009	0.002	-0.978	0.145
$\boldsymbol{\Omega^e_5}$	2.087	-1.015	-0.791	0.202	0.243	-0.079	-0.048	-0.009	3.942	-0.747	221.322	-34.247
	-0.410	0.190	0.170	-0.040	-0.063	0.020	0.007	0.002	-0.748	0.144	-41.681	6.573
$\boldsymbol{\Omega^e_6}$	155.399	-78.526	-27.519	7.911	20.548	-3.308	-0.060	-0.978	221.322	-41.681	17377.76	-2272.88
	-24.672	11.218	6.736	-1.347	-3.676	0.912	0.103	0.145	-34.247	6.573	-2272.88	329.277
